# Cyclic adenosine monophosphate potentiates immune checkpoint blockade therapy in acute myeloid leukemia

**DOI:** 10.1002/ctm2.1489

**Published:** 2023-11-23

**Authors:** Ping Mao, Wenbin Feng, Zongmeng Zhang, Changhao Huang, Sujin Zhou, Zhenggang Zhao, Yunping Mu, April Yuanyi Zhao, Lina Wang, Fanghong Li, Allan Z. Zhao

**Affiliations:** ^1^ Department of Biomedical and Pharmaceutical Sciences Guangdong University of Technology Guangdong China

Dear Editor,

The response rates to immune checkpoint blockade therapies (ICBs) are extremely low in acute myeloid leukemia (AML).^1^ Additional pharmacological agents are needed to enhance the efficacy of ICBs for AML patients. The anti‐tumorigenic inflammation is known to play a key role in the outcomes of ICBs.^2^, ^3^ Cyclic adenosine monophosphate (cAMP) has often been associated with anti‐inflammatory effects such as diminishing the levels of pro‐inflammatory cytokines.^4^ However, recent reports have highlighted a positive role of cAMP in immune cell function and homeostasis,^5^, ^6^, ^7^ particularly its role in ICBs remains to be defined. Our study proposes that cAMP can offer therapeutic benefit in ICBs and that combining cAMP‐elevating agents with programmed death‐ligand 1 monoclonal antibody (PD‐L1 mAb) can enrich circulating CD8^+^ T cells, and significantly prolong the survival of AML model mice.

We first analyzed several cancer cohorts treated with ICBs from the GEO database. Gene set enrichment analysis (GSEA) indicated that the cAMP pathway (544 genes from Pathway Unification Database, Table S1) was significantly elevated in the responders of ICBs among non‐small cell lung cancer patients compared to that in the non‐responders (Figure 1A). Similar results were also observed in renal cell carcinoma and thymic carcinoma patients (Figure 1A), and even in mouse renal cell carcinoma and mouse triple‐negative breast cancer (TNBC) samples (Figure S1A,B). Interestingly, we also saw an upward trend of the cAMP pathway on day 3 and a significant increase on day 7 after ICB treatment compared with that of pretreated TNBC mice (Figure S1C), suggesting that the high response to ICBs correlates with increased cAMP pathway. Kaplan‐Meier survival analysis further showed that cancer patients with high cAMP signature (10 genes from Molecular Signatures Database, adenylyl cyclase [ADCY] types 1–10) expression had a better prognosis in patients with glioblastoma, melanoma, and esophageal adenocarcinoma treated with ICBs than those patients with low cAMP signature (Figure 1B–D and Figure S1D,E). Thus, elevation of cAMP can potentially offer therapeutic benefit in ICB‐treated patients.

**FIGURE 1:**
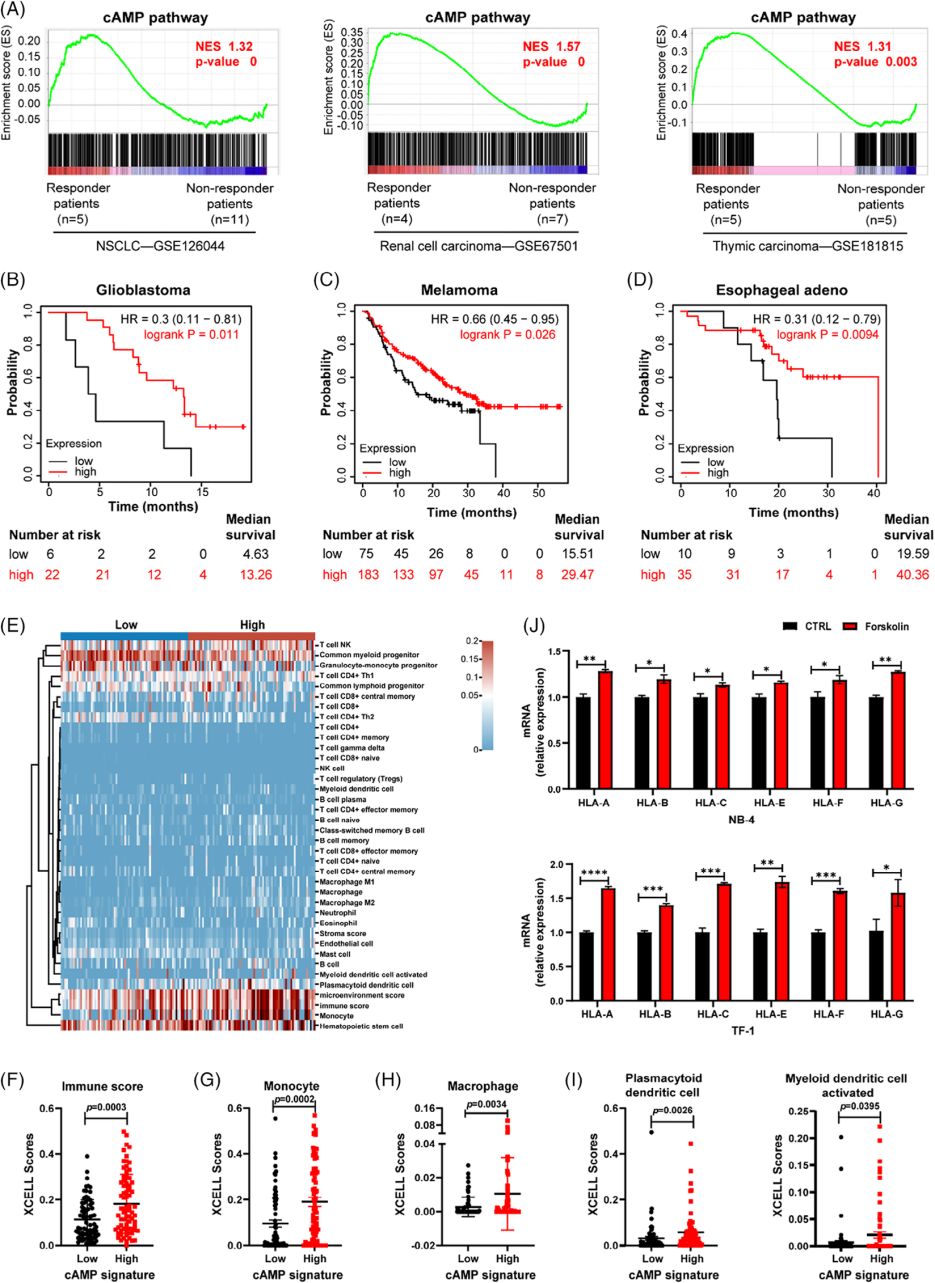
High response and prolonged survival benefit of immune checkpoint blockade therapies (ICBs) correlate with upregulated cyclic adenosine monophosphate (cAMP) pathway and cAMP signature. (A) Gene set enrichment analysis (GSEA) of cAMP pathway was performed from transcriptomes of the responder patients (red) versus the non‐responder patients (blue) to ICBs. Immunotherapy: anti‐PD1 therapy (Responder: CR and PR, non‐responder: SD and PD. CR, complete response; PR, partial response; SD, stable disease; PD, progressive disease). (B) Overall survival (OS) curves for high and low cAMP signature expression of glioblastoma cohorts after anti‐PD‐1 treatment. (C) OS curves for high and low cAMP signature expression of melanoma cohort after the anti‐PD‐1 treatment. (D) OS curve for high and low cAMP signature expression of oesophagal adenocarcinoma cohorts in the anti‐programmed death‐ligand 1 (anti‐PD‐L1) treatment cohort. Data are analyzed using the Kaplan‐Meier Plotter database (http://kmplot.com/analysis/index.php?p = service&cancer = immunotherapy). (E) Immune cell score heatmap, different colors represent different expression distribution in low/high cAMP signature expression groups (*n* = 150 total, 75/group). The immune score (F), monocyte score (G), macrophage score (H), and dendritic cell score (I) in low/high cAMP signature groups analyzed by XCELL algorithm. (J) Quantitative real‐time polymerase chain reaction (qRT‐PCR) of HLA‐A/B/C/E/F/G genes in the NB4 and TF‐1 cells treated with dimethylsulfoxide (DMSO) or forskolin (24 μM, 24 h, *n* = 3). Data are shown as mean ± SD. In F‐J, *p*‐values are from Wilcoxon test, in (K), *p*‐values are from a two‐sided unpaired *t*‐test. *p < .05, **p < .01, ***p < .001, ****p < .0001.

XCELL algorithm analysis of the Cancer Genome Atlas (TCGA)‐AML data showed that the high cAMP signature group had a higher immune score (the sum of all immune cell scores) than the low cAMP signature group (Figure 1E–F). Further analysis indicated that high cAMP signature groups were positively correlated with increased scores of monocytes, macrophages and dendritic cells (Figure 1G–I). These immune cells play a crucial role in antigen presentation. GSEA further showed that antigen processing and presentation pathways were increased in the high cAMP signature group (Figure S1F). Besides, in response to forskolin (an ADCY activator, which can increase intracellular cAMP levels), the expression of class I MHC (HLA‐A/B/C/E/F/G) significantly increased in HL‐60, NB‐4 and TF‐1 cells (Figure 1J and Figure S1G). These data suggested that cAMP promotes antigen presentation in AML cells, which in turn should favour ICBs.

Fluorescence‐activated cell sorting (FACS) analysis (Figure 2A,B), quantitative real‐time polymerase chain reaction (Figure S2) and Western blot assays (Figure 2C,D) all demonstrated that cAMP‐induced PD‐L1 expression. Understanding the molecular mechanisms of PD‐L1 regulation is important for improving the efficacy of ICBs. cAMP regulates the expression of numerous genes via the ATF/CREB family.^8^, ^9^ Correlation analysis revealed the strongest positive correlation between PD‐L1 expression and ATF2 expression among ATF/CREB family members in adult de novo AML patients and pediatric AML patients (Figure 2E and Figure S3A–C). Interestingly, correlation analysis of the TCGA database also showed that ATF2 expression was positively correlated with PD‐L1 expression in multiple (up to 29) cancer types (Figure 2F, FigureS4A,B). We initially confirmed that forskolin significantly increased ATF2 phosphorylation (at Thr197) in NB‐4 and TF‐1 cells (Figure 2G). Secondly, we demonstrated that ATF2‐knockdown (sh‐*ATF2*) significantly decreased PD‐L1 expression (Figure 2H,I). In contrast, ATF2‐overexpression (*ATF2*‐OE) stimulated PD‐L1 expression (Figure 2J,K). Furthermore, the results of the CUT and RUN assay confirmed that the p‐ATF2 protein could directly bind to the PD‐L1 gene and stimulate PD‐L1 mRNA expression in NB‐4 and TF‐1 cells. (Figure 3A). Similar results were also observed in forskolin‐treated cells than in the control (dimethylsulfoxide [DMSO]) (Figure 3B). Three binding sites of ATF2 were identified on the PD‐L1 promoter through the JASPER database. We generated promoter constructs containing mutations in these three regions that would disrupt the binding of p‐ATF2 (Figure 3C). In a dual luciferase assay, the 293T cells transfected with ATF2 plasmid significantly elevated PD‐L1 expression than the control plasmid‐transfected cells (Figure 3D). Mutating the ATF2 binding sites in the PD‐L1 promoter completely nullified ATF2‐stimulated luciferase activity (Figure 3D). Similar results were observed in forskolin‐treated cells (Figure 3E). Collectively, we revealed that ATF2 is a major positive regulator of PD‐L1 mRNA expression by binding directly to the PD‐L1 promoter in AML cells.

**FIGURE 2:**
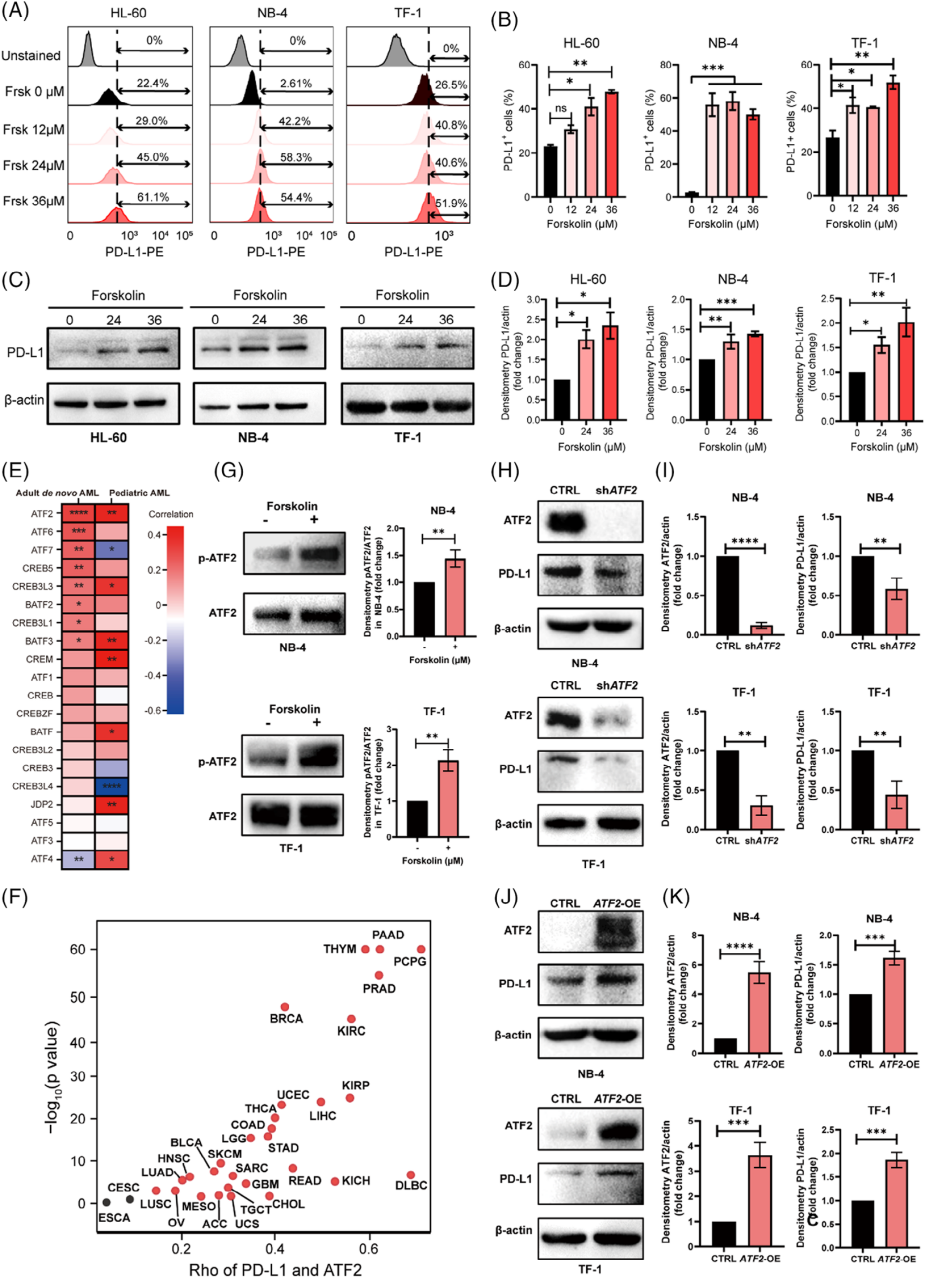
ATF2 functions as a cyclic adenosine monophosphate (cAMP) downstream target in the regulation of programmed death‐ligand 1 (PD‐L1) transcription in acute myeloid leukaemia (AML) cells. (A) Fluorescence‐activated cell sorting (FACS) analysis of cell‐surface expression of PD‐L1 in three different AML cell lines treated with either dimethylsulfoxide (DMSO) or forskolin (24 h). (B) Quantity analysis of PD‐L1^+^ cells (%). (C) Western blot assay of PD‐L1 protein expression in the AML cell lines treated with DMSO or forskolin (24 h). (D) Quantitative analysis of PD‐L1 protein expression. (E) The heatmap of PD‐L1 correlation with various CREB/ATF family members in adult de novo AML (*n* = 173) and pediatric AML patients (*n* = 45). Red, positive correlation. Blue, negative correlation. (F) Spearman correlation analysis of PD‐L1 and ATF2 from TCGA database. The red dots indicate *p*‐values < .05. (G) AML cell lines are treated with forskolin (24 μM, 5 min). Phospho‐ATF2 (Thr197) and total ATF2 expression were analyzed by Western blot. (H, I) NB‐4 and TF‐1 cells were transfected with sh‐*ATF2* or CTRL shRNA lentivirus. ATF2 and PD‐L1 expression was analyzed by Western blot (H) and subsequent quantification (I). (J, K) NB‐4 and TF‐1 cells were transfected with *ATF2*‐OE or negative control lentivirus. ATF2 and PD‐L1 expression was analyzed by Western blot (J) and subsequent quantification (K). Data are shown as mean ± SD, n = 3. In (B) and (D), *p*‐values are from one‐way analysis of variance (ANOVA) analysis, followed with Tukey test. In (G), (I) and (K), *p*‐values are from a two‐sided unpaired *t*‐test. ns, no significance, *p < .05, **p < .01, ***p < .001, ****p < .0001.

**FIGURE 3:**
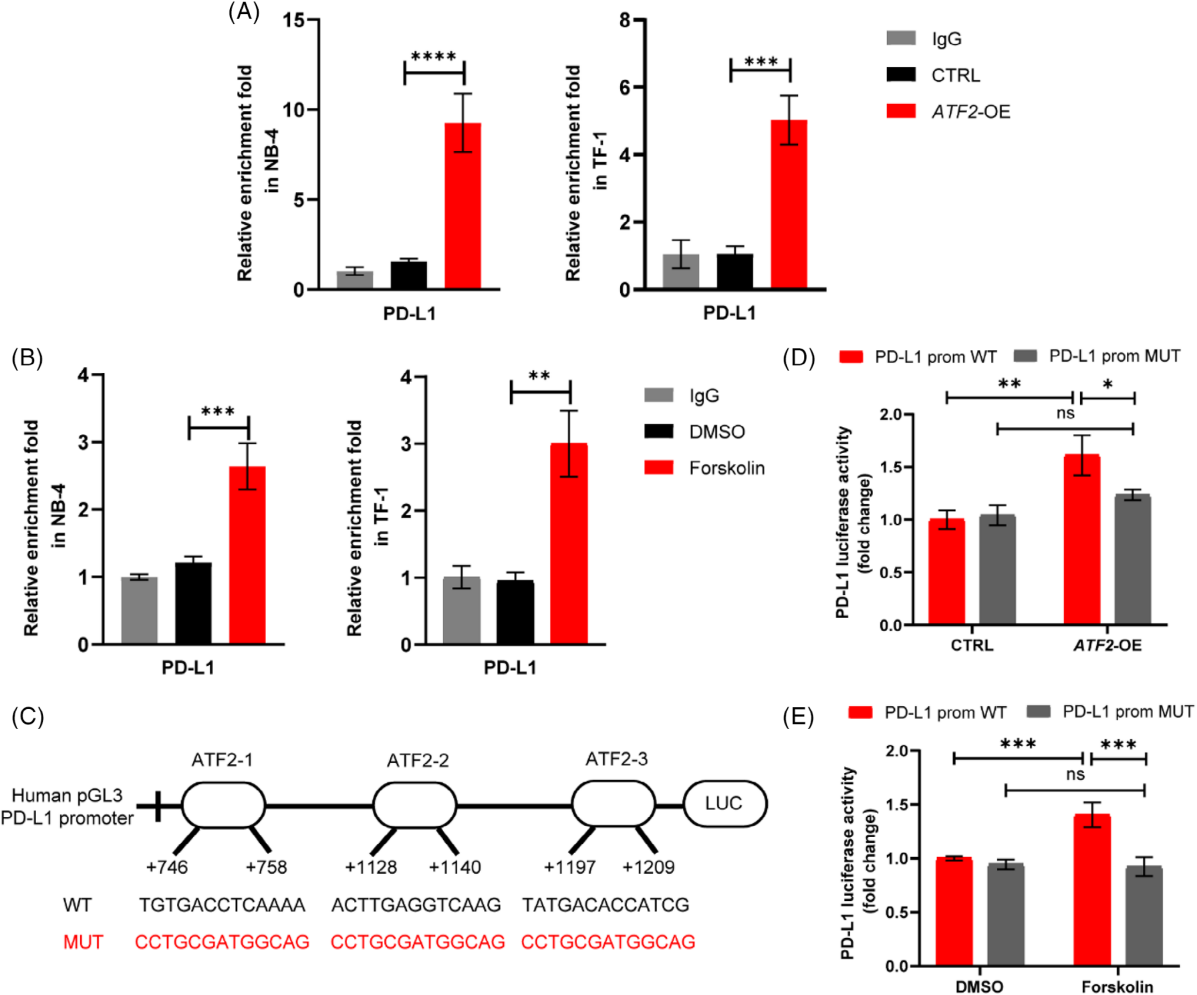
ATF2 binds directly to the programmed death‐ligand 1 (PD‐L1) promoter. (A and B) ATF2 binding to the PD‐L1 promoter was determined by CUT&RUN‐quantitative real‐time polymerase chain reaction (qRT‐PCR) in acute myeloid leukaemia (AML) cell lines (NB‐4 and TF‐1) transfected with *ATF2*‐OE lentivirus (A) or treated with forskolin (B). (C) Schematic representation of the PD‐L1 promoter cloned into the pGL3 vector. Three predicted ATF2 binding motifs are shown and promoter constructs containing mutations in these three regions to cause ATF2‐binding deficiency are generated. The predicted ATF2 binding motifs of PD‐L1 were using JASPAR (http://jaspar.genereg.net/). (D, E) Analysis of PD‐L1 WT or mutant promoter activity in AML cell lines (NB‐4 and TF‐1) transfected with *ATF2*‐OE plasmid (D) or treated with forskolin (E). Data are shown as mean ± SD, *n* = 3. In (A) and (B), *p*‐values are from a two‐sided unpaired *t*‐test. In (D) and (E), *p*‐values are from two‐way analysis of variance (ANOVA) analysis. ns, no significance, *p < .05, **p < .01, ***p < .001, ****p < .0001.

Finally, we deployed an AML‐mouse model derived from the MLL‐AF9 cells with the experimental design shown in Figure 4A. FACS analysis of the blood samples indicated that there were no significant differences between CD3^+^ and CD4^+^ T cell populations among all treated AML mice (Figure 4B,C and Figure S5A,B). The proportion of CD8^+^ T cells in the PD‐L1 mAb treated group, however, was significantly higher than that in the control group. Co‐treatment of apremilast (an FDA‐approved PDE4 inhibitor) and PD‐L1 mAb further elevated the CD8^+^ T cell population (Figure 4D and Figure S5B). Importantly, apremilast‐treatment increased PD‐L1 expression in AML mice (Figure S5C). On day 15, there was no significant difference in GFP^+^‐AML cells (indicative of AML proliferation) among the CTRL, apremilast, and PD‐L1 mAb treated groups. However, GFP^+^‐AML cells were significantly reduced in the combinatorial treatment group (Figure 4E,F). On day 21, a significant reduction of GFP^+^‐AML cells appeared in the PD‐L1 mAb‐treated mice, and the combinatorial treatment further decreased GFP^+^‐AML cell population (Figure 4E,G). Importantly, only the co‐treatment modality significantly prolonged the survival of AML mice compared to the CTRL and the PD‐L1 mAb treated group (Figure 4H). And none of the drug‐treated mice exhibited weight loss relative to the CTRL (Figure S5D). Except for increasing cAMP levels, apremilast has a powerful anti‐inflammatory effect,^10^ which also plays a key role in the outcome of ICBs.^2^, ^3^

**FIGURE 4:**
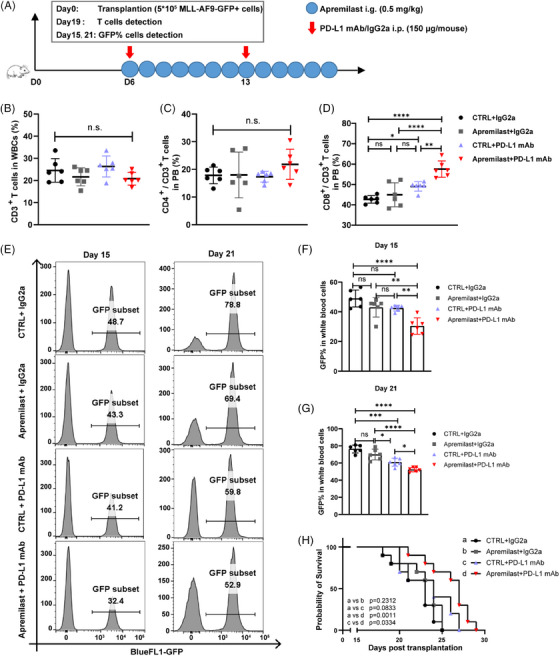
Synergistic effect of apremilast and programmed death‐ligand 1 (PD‐L1) mAb therapy in MLL‐AF9‐derived acute myeloid leukaemia (AML) model. (A) A schematic timeline view of the treatment plan. (B) Fluorescence‐activated cell sorting (FACS) quantification of CD3^+^ in white blood cells from MLL‐AF9‐driven AML mice. (C) FACS quantification of CD4^+^ in CD3^+^ from MLL‐AF9‐driven AML mice. (D) FACS quantification of CD8^+^ in CD3^+^ from MLL‐AF9‐driven AML mice. (E) Representative flow cytometry histograms of GFP^+^‐AML cells in the white blood cells on days 15 and 21. (F, G). Quantification of the GFP^+^‐AML cells in the WBCs on day 15 (F) and on day 21 (G) (*n* = 6). (H) The statistical analysis of survival rate of mice (*n* = 40 total, 10/treatment arm). Data are shown as mean ± SD. In (B)–(D), (F) and (G), *p*‐values are from one‐way analysis of variance (ANOVA) analysis, followed with Tukey test. In H, *p*‐values are from log‐rank (Mantel‐Cox) test. ns, no significance, *p < .05, **p < .01, ***p < .001, ****p < .0001.

## 1 CONCLUSIONS

This study revealed a novel molecular mechanism whereby cAMP induces PD‐L1 expression through the transcription factor ATF2. Combining a cAMP‐elevating agent with PD‐L1 mAb‐enriched circulating CD8^+^ T cells, and significantly prolonged the survival of AML model mice. Such a combinatorial strategy can be clinically trialled as a promising modality for AML patients.

## AUTHORSHIP CONTRIBUTIONS

Ping Mao: Conceptualization, Methodology, Validation, Writing–original draft. Wenbin Feng, Zongmeng Zhang and Changhao Huang: Validation. Sujin Zhou and Zhenggang Zhao: laboratory supervision. Yunping Mu: Project supervision. April Yuanyi Zhao: experimental assistance & data analysis. Lina Wang: laboratory assistance & supervision. Fanghong Li and Allan Z. Zhao: Conceptualization, Resources, Writing—review & editing, Funding acquisition.

## CONFLICT OF INTEREST STATEMENT

The authors declare no conflict of interest.

## Supporting information

Supporting Information

Supporting Information

## Data Availability

All data generated or analyzed during this study are included in this published article.
