# Supplementary material for: Cyclic adenosine monophosphate potentiates immune checkpoint blockade therapy in acute myeloid leukemia
Source: Clin Transl Med. 2023 Nov 23;13(11):e1489. doi: 10.1002/ctm2.1489 (PMC10667622; doi:10.1002/ctm2.1489)
Supplement: Supplementary file 1 — Supporting Information [file CTM2-13-e1489-s001.docx]

Supplemental table 1—cAMP pathway

Supplemental table 2—oligonucleotides sequences

**
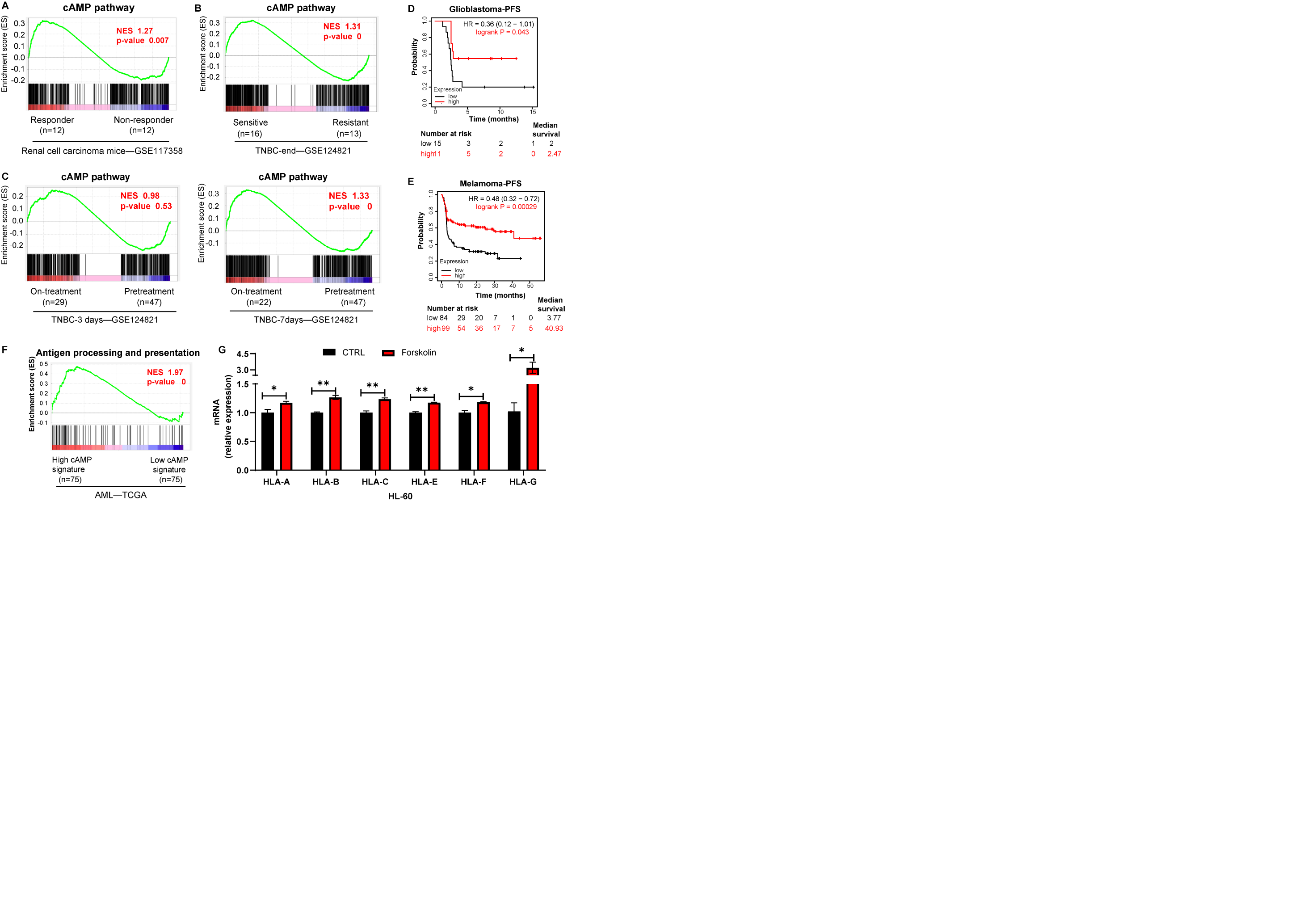
**

**Supplemental Figure 1. High response and prolonged survival benefit of ICBs correlate with upregulated cAMP pathway and cAMP signature.** **(A)** GSEA of cAMP pathway was performed from transcriptomes of the responder (red) versus the non-responder (blue) to ICBs in Renal cell carcinoma-xenografted mice. Immunotherapy: anti-PD1 and anti-CTLA-4 combination therapy (Responder: CR and PR, non-responder: SD and PD, CR, complete response; PR, partial response; SD, stable disease; PD, progressive disease). **(B)** GSEA of cAMP pathway was performed from transcriptomes of the sensitive (red) versus the resistant (blue) to ICBs in TNBC-xenografted mice. Immunotherapy: anti-PD1 and anti-CTLA-4 combination therapy (Responder: CR and PR, non-responder: SD and PD, CR, complete response; PR, partial response; SD, stable disease; PD, progressive disease). **(C)** GSEA of cAMP pathway was performed from transcriptomes of on-treatment of ICBs (on day 3, 7) (red) versus that of the pretreated (blue) in TNBC-xenografted mice. Immunotherapy: anti-PD1 and anti-CTLA-4 combination therapy. **(D)** PFS curves for high and low cAMP signature expression of glioblastoma cohorts after anti-PD-1 treatment. **(E)** PFS curves for high and low cAMP signature expression of melanoma cohort after the anti-PD-1 treatment. **(F)** GSEA of antigen processing and presentation pathway was performed from transcriptomes of high cAMP signature (red) versus low cAMP signature (blue) in AML patients. **(G)** Quantitative-RT-PCR of HLA-A/B/C/E/F/G genes in HL-60 cells treated with DMSO or forskolin (24μM, 24h). Data are shown as mean ± SD in triplicates. In G, *p* values are from two-sided unpaired t test. **p* < 0.05, ***p* < 0.01.


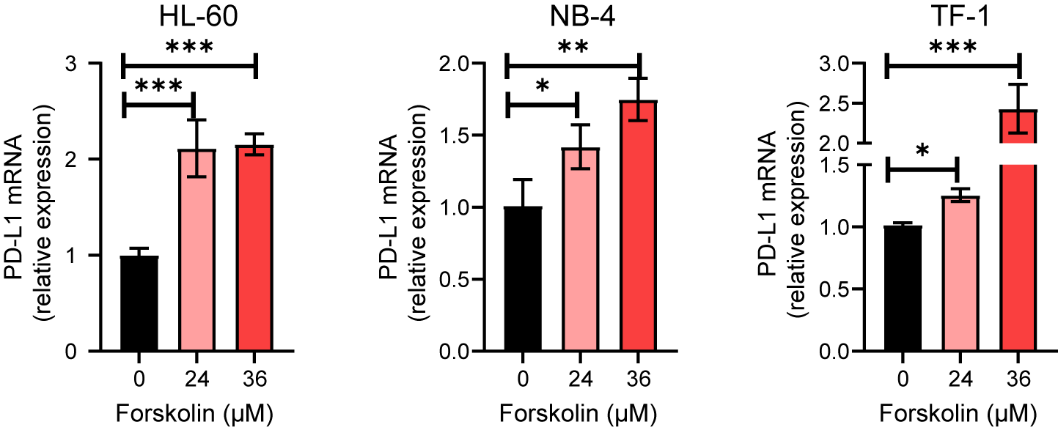


**Supplemental Figure 2.** Quantitative RT-PCR assay of PD-L1 mRNA in the AML cell lines treated with DMSO or forskolin (6-16h). Data are shown as mean ± SD, n=3. *p* values are from one-way ANOVA analysis, followed with Tukey test. **p* < 0.05, ***p* < 0.01, ****p* < 0.001.


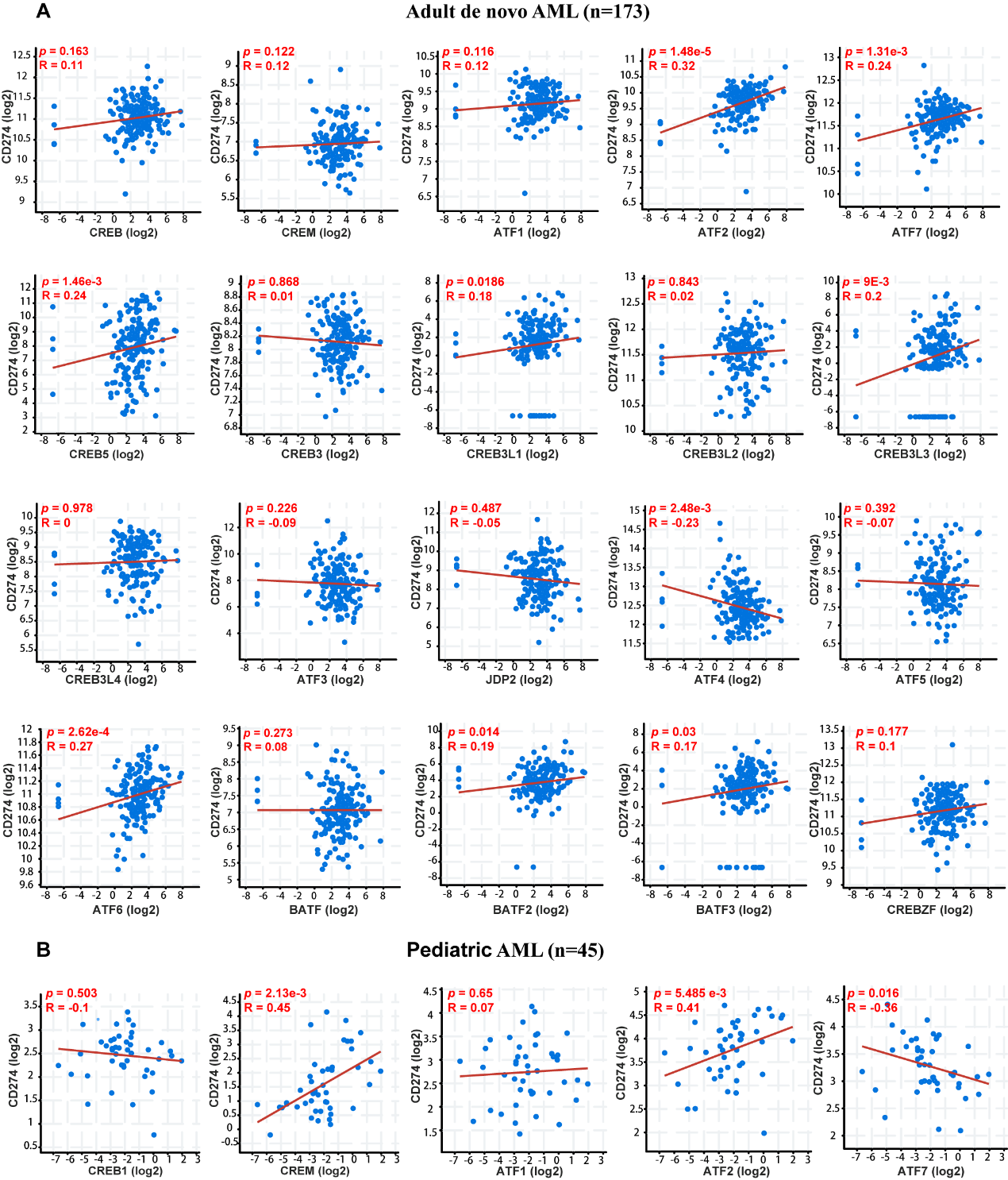


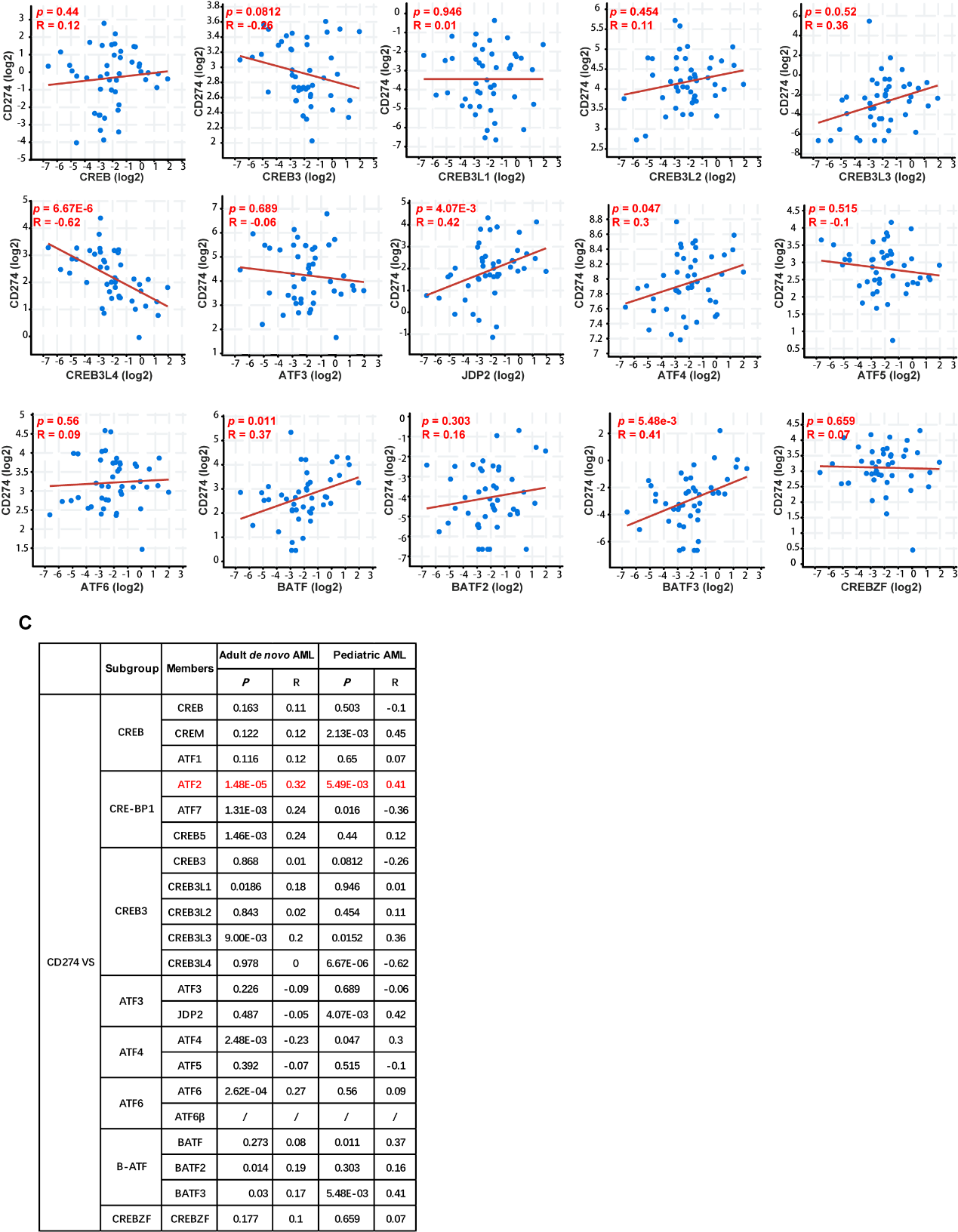


**Supplemental Figure 3. The Spearman correlation of PD-L1 and CREB/ATF family members in AML patients. (A)** Spearman correlation between PD-L1 (CD274) and CREB/ATF family in adult de novo AML (n=173). **(B)** Spearman correlation between PD-L1 (CD274) and CREB/ATF family in pediatric AML (n=45). **(C)** Summary of correlation between PD-L1 (CD274) and CREB/ATF family in adult de novo AML and pediatric AML. Data are form the TCGA and TARGET collection and analyzed in the cBioPortal platform (https://www.cbioportal.org/), and data for ATF6β were not available.


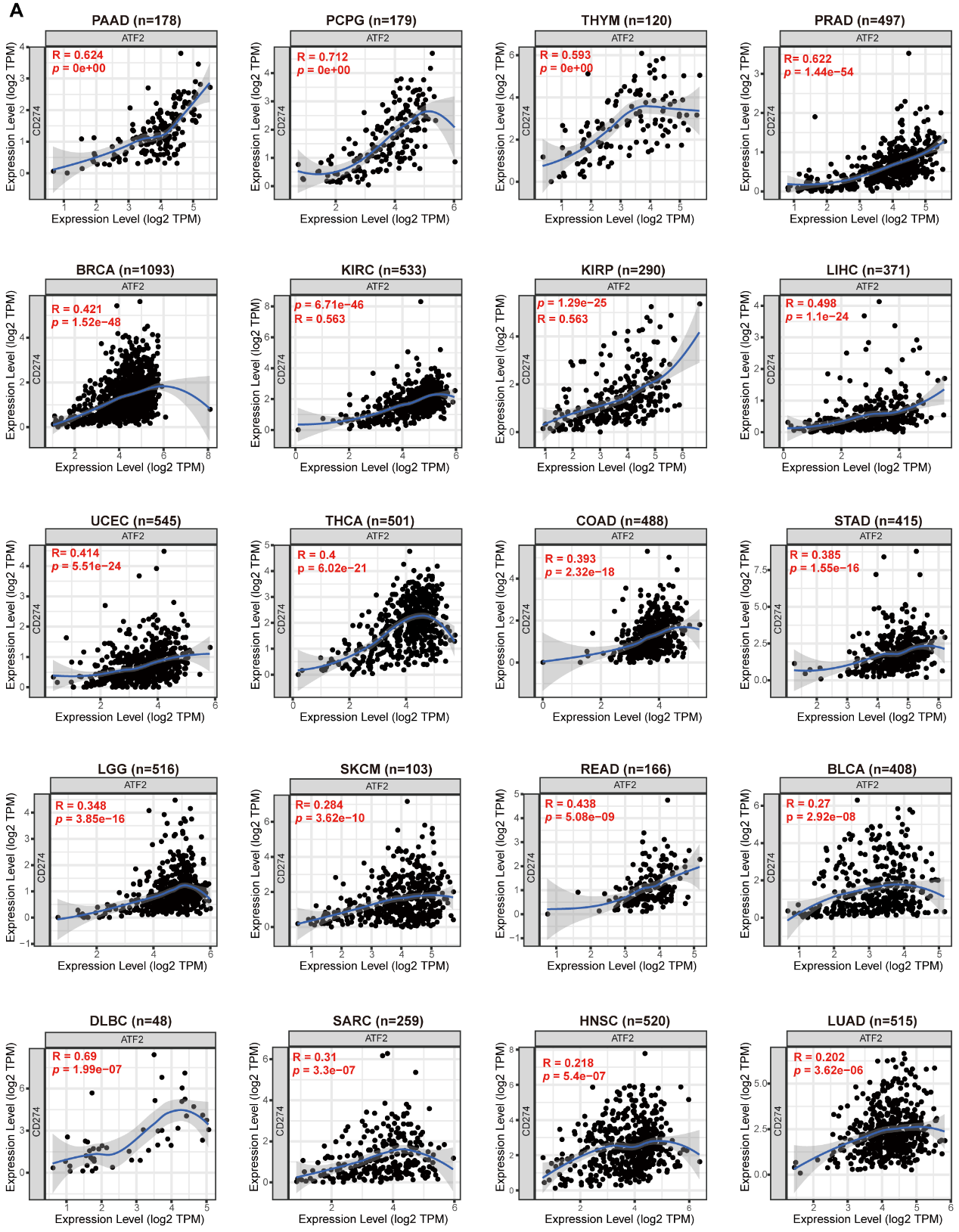


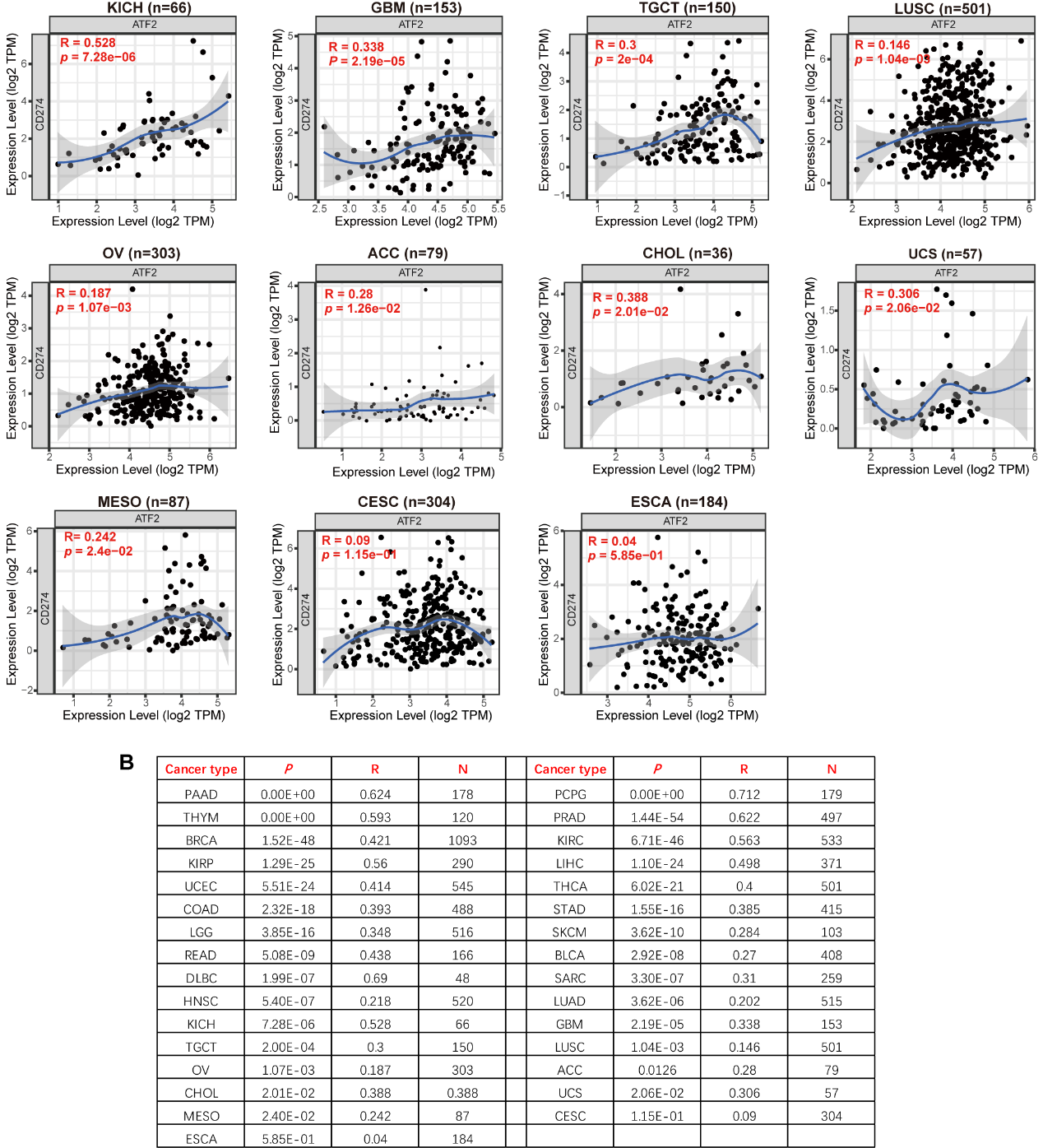


**Supplemental Figure 4. Spearman correlation analysis of PD-L1 and ATF2 in cancers. (A)** Spearman correlation between PD-L1 (CD274) and ATF2 in 31 types of cancers. **(B)** Summary of correlation between PD-L1 (CD274) and ATF2 in 31 types of cancers. Data are form the TCGA collection and analyzed in the TIMER platform (https://cistrome.shinyapps.io/timer/).


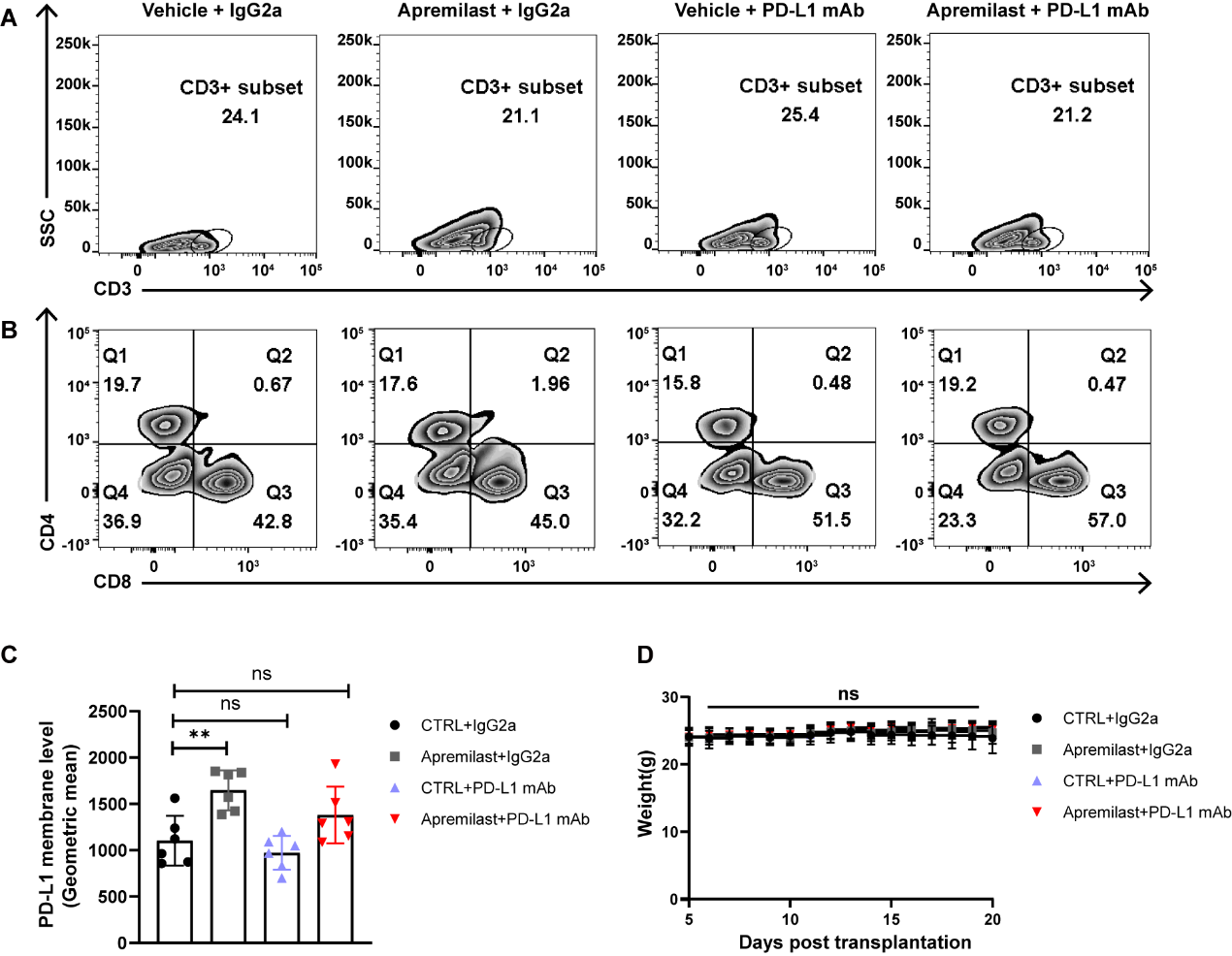


**Supplemental Figure 5.** **Synergistic effect of apremilast and PD-L1 mAb therapy in MLL-AF9-derived AML model. (A)** Representative zebra plots showing the CD3^+^ T cells population in white blood cells from MLL-AF9-driven AML mice**. (B)** Representative zebra plots showing the CD4^+^ T and CD8^+^ T cells population in CD3^+^ T cells from MLL-AF9-driven AML mice**. (C)** Geometric mean fluorescence intensity of PD-L1 in GFP+-AML cells from MLL-AF9-driven AML mice (n=6). **(D)**. The body weight of the mice during treatment. Data shown are mean ±SD. In C, *p* values are from one-way ANOVA with Tukey’s multiple comparisons test. In E, *p* values are from two-way ANOVA analysis. ns, no significance, ** p<0.01.
