# Supplementary material for: Cyclic adenosine monophosphate potentiates immune checkpoint blockade therapy in acute myeloid leukemia
Source: Clin Transl Med. 2023 Nov 23;13(11):e1489. doi: 10.1002/ctm2.1489 (PMC10667622; doi:10.1002/ctm2.1489)
Supplement: Supplementary file 2 — Supporting Information [file CTM2-13-e1489-s002.docx]

**Supplementary Materials and Methods**

**Data collection and processing**

Patients’ RNA sequencing data and corresponding clinical follow-up information were downloaded from the publicly available database--The Cancer Genome Atlas (TCGA) (<https://portal.gdc.cancer.gov>) and the Gene Expression Omnibus (GEO) database (GSE126044, GSE67501, GSE181815, GSE124821 and GSE117358) (<https://www.ncbi.nlm.nih.gov/geo/>). Gene Set Enrichment Analysis (GSEA) was performed using GSEA 4.3.2 software and the “cAMP pathway” curated gene sets from the Pathway Unification Database, “Antigen processing and presentation” curated gene sets from the Molecular Signatures Database [1]. The OS and PFS data of anti-PD-1/PD-L1 treated cancer patient were obtained from the Kaplan-Meier Plotter database (<http://kmplot.com/analysis/index.php?p=service&cancer=immunotherapy>) [2]. The analysis was constructed by the R v4.0.3 software package ggplot2 (v3.3.3). The correlation analysis between PD-L1 and ATF/CREB family in adult *de novo* AML and pediatric AML patients used the cBioPortal database (<https://www.cbioportal.org/>) [3]. The correlation analysis between PD-L1 and ATF2 in 31 types of tumors was based on the TIMER database (<https://cistrome.shinyapps.io/timer/>) [4].

**Quantifying the Tumor Microenvironment**

The cellular abundance of 64 immune and stromal cells was calculated for 150 AML patients in the TCGA datasets by using the XCELL algorithm [5]. The principle of the algorithm is to extract the signatures of 64 immune cells and stromal cells by using a machine learning algorithm and convert the enrichment scores into cell-type scores. Compensation corrections were finally made for closely related cell-type fractions. All these analytic methods and R package were implemented by R (v4.0.3) foundation for statistical computing and software packages ggplot2 (v3.3.3) and pheatmap.

**Cell lines**

AML cell lines HL-60, NB-4, and TF-1 were cultured as described previously [6]. The identity of the cell lines was confirmed by STR profiling and the cell lines were regularly tested for mycoplasma contamination. All cell lines were pre-existent in the investigator’s laboratory and/or obtained from [www.cellbank.org.cn](http://www.cellbank.org.cn).

**RNA isolation, cDNA synthesis and quantitative-RT-PCR**

RNA was isolated from AML cell lines using Total RNA Kit (Omega, R6834-01). One microgram of RNA was used for cDNA synthesis using a High-Capacity cDNA Reverse Transcription Kit (Vazyme, Q711-02). The levels of transcripts were measured on a QuantStudio 5 real-time PCR system (Applied Biosystems) using iTaq Universal SYBR Green Supermix (Vazyme, R223-01). Relative gene expression was calculated using the 2^–ΔΔCT^ method. The oligonucleotide sequences are listed in supplemental table 2.

**Fluorescence-activated cell sorting (FACS) analysis**

Six mice were randomly selected from each group, Mouse anticoagulant blood samples were obtained, and erythrocytes were lysed (Cat. no: 555899, BD). AML (GFP^+^) cells were detected directly by flow cytometry in an FITC channel gated. After blocking with CD16/CD32 (BD, 553141) antibody, the white blood cells in peripheral blood samples were stained with anti-mouse PD-L1-APC (Biolegend, 124312), anti-mouse CD3-APC-Cy7 (BD, 557596), anti-mouse CD4-PE (BD, 553652), and anti-mouse CD8-PerCP-Cy5.5 (BD, 551162) antibodies in the dark for 30 min before analysis. Stained cells were analyzed by FACS Dxp AthenaTM (Cytek, USA). Data were further analyzed by Flow Jo v10.6.2. software.

AML cells were incubated with forskolin (Beyotime, S1612) for 24 h. Afterward, the cells were harvested and washed with PBS and then stained with anti-human PD-L1-PE antibody (BD, 557924) in the dark for 15 min before analysis.

**Immunoblotting analysis**

Western blot assays were performed to examine PD-L1, ATF2, phospho-ATF2 (Thr197) in AML cells, as described previously [6]. Anti-PD-L1 (1:1000, 13684S), anti-ATF2 (1:1000, 35031S), and anti-phospho-ATF2 (Thr197) (1:1000, 24329S）were from Cell Signaling Technology.

**Lentiviral transduction**

*ATF2*-shRNA and *ATF2*-OE lentiviral vector carrying hairpins were purchased from Shanghai Genechem. The targeting sequences were as follows: sh*ATF2*: gcATCATTACAGGTTCCCAAT. NB-4/TF-1 cells with 80% confluence were transfected using HitransG P/HitransG A (Genechem) for 24-48 h and selected by puromycin (NB4:0.5 ng/ml, TF-1:1.5 ng/ml).

**Dual Luciferase Reporter Assay**

293T cells were seeded in 24-well plates with a confluence rate of 70-80% and then transfected using the Lipofectamine™3000 Transfection Reagent HD (Thermo Fisher, L3000001) according to the manufacturer’s instruction. All these Renilla, *ATF2*-OE, PD-L1-WT and PD-L1-MUT promoter plasmid were synthesized by Shanghai Genechem. Forskolin (30 μM) was added after overnight transfection. Relative luciferase units (RLUs) were measured using the Dual-Glo Luciferase Assay System (Genecopoeia, LF001) according to the manufacturer’s instructions. RLUs from firefly luciferase signal were normalized by RLUs from Renilla signal.

**CUT & RUN**

CUT&RUN was performed on 3 × 10^5^ NB-4/TF-1 cells transfected with *ATF2*-OE and control (CTRL) lentivirus vector or treated with forskolin and DMSO using CUT&RUN Assay Kit (Vazyme, HD101) as per manufacturer's protocol. In brief, AML cells were bound to Concanavalin A beads, permeabilized with digitonin, and incubated with p-ATF2 antibody (Cell Signaling Technology, 24329S; 1:50) and anti-IgG (Cell Signaling Technology, 2729S, 1:50) overnight at 4°C on a thermo shaker. On the next day, Protein A-fused micrococcal nuclease was incubated for 60 min at 4°C to bind IRF1 antibody and digest bound sites, followed by incubation at 37°C for 10 min to release digested DNA fragments. SYBR Green quantitative-RT-PCR was performed using the human PD-L1 primers: forward primer: 5’-GCTGCACTAATTGTCTATTGGGA-3’; reverse primer: 5’-AATTCGCTTGTAGTCGGCACC-3’.

**MLL-AF9-induced acute myeloid leukemia mouse model**

Six-week-old male C57BL/6 mice were purchased from the GemPharmatech. Liquid nitrogen preserves murine AML primary spleen cells with a report green fluorescent protein (GFP) initiated by human MLL-AF9 fusion protein were kindly provided by Professor Cheng Tao from State Key Laboratory of Experimental Hematology, China [7]. 5×10^5^ murine MLL-AF9-GFP+-AML primary spleen cells were transplanted into C57BL/6 mice via tail vein injection. On day 6 after transplantation, mice were selected and assigned to groups randomly, the recipient mice received apremilast (0.5 mg/kg) or vehicle control (40 % Hydroxypropyl-β-cyclodextrin + 6 % HS-15 + 5 % DMSO in saline) daily via oral administration, and/or with anti-mouse PD-L1 (BioXcell, BE0101) or rat IgG2b isotype isotype control (BioXcell, BE0090), administered intraperitoneally (IP, 150 μg per injection) at a 7-day interval. All mice were maintained under specific pathogen-free conditions. All animal experimental protocols were approved by the Experimental Animal Ethics Committee of Guangdong Pharmaceutical University.

**Statistical analyses**

Statistical analyses were performed using GraphPad Prism (version 9.0.0) and R software (v4.0.3) and *p*<0.05 was considered statistically significant.

**References**

[1] A. Subramanian, P. Tamayo, V.K. Mootha, et al., Gene set enrichment analysis: a knowledge-based approach for interpreting genome-wide expression profiles, Proc Natl Acad Sci U S A, 102 (2005) 15545-15550.<http://dx.doi.org/10.1073/pnas.0506580102>

[2] S.A. Kovacs, B. Gyorffy, Transcriptomic datasets of cancer patients treated with immune-checkpoint inhibitors: a systematic review, J Transl Med, 20 (2022) 249.<http://dx.doi.org/10.1186/s12967-022-03409-4>

[3] J. Gao, B.A. Aksoy, U. Dogrusoz, et al., Integrative analysis of complex cancer genomics and clinical profiles using the cBioPortal, Sci Signal, 6 (2013) pl1.<http://dx.doi.org/10.1126/scisignal.2004088>

[4] T. Li, J. Fan, B. Wang, et al., TIMER: A Web Server for Comprehensive Analysis of Tumor-Infiltrating Immune Cells, Cancer Res, 77 (2017) e108-e110.<http://dx.doi.org/10.1158/0008-5472.CAN-17-0307>

[5] D. Aran, Z. Hu, A.J. Butte, xCell: digitally portraying the tissue cellular heterogeneity landscape, Genome Biol, 18 (2017) 220.<http://dx.doi.org/10.1186/s13059-017-1349-1>

[6] P. Mao, C. Huang, Y. Li, et al., Pharmacological targeting of type phosphodiesterase 4 inhibits the development of acute myeloid leukemia by impairing mitochondrial function through the Wnt/beta-catenin pathway, Biomed Pharmacother, 157 (2023) 114027.<http://dx.doi.org/10.1016/j.biopha.2022.114027>

[7] Y. Liu, H. Cheng, S. Gao, et al., Reprogramming of MLL-AF9 leukemia cells into pluripotent stem cells, Leukemia, 28 (2014) 1071-1080.<http://dx.doi.org/10.1038/leu.2013.304>
